# Fluorescence confocal endomicroscopy of the cervix: pilot study on the potential and limitations for clinical implementation

**DOI:** 10.1117/1.JBO.21.12.126011

**Published:** 2016-12-21

**Authors:** Colin Schlosser, Nico Bodenschatz, Sylvia Lam, Marette Lee, Jessica N. McAlpine, Dianne M. Miller, Dirk J. T. Van Niekerk, Michele Follen, Martial Guillaud, Calum E. MacAulay, Pierre M. Lane

**Affiliations:** a British Columbia Cancer Research Centre, Department of Integrative Oncology, 675 West 10th Avenue, Vancouver, British Columbia V5Z 1L3, Canada; b University of British Columbia, Division of Gynecologic Oncology, 2775 Laurel Street, Vancouver, British Columbia V5Z 1M9, Canada; c British Columbia Cancer Agency, Department of Pathology, 600 West 10th Avenue, Vancouver, British Columbia V5Z 4E6, Canada; d Brookdale University Hospital and Medical Center, One Brookdale Plaza, Brooklyn, New York 11212, United States

**Keywords:** endomicroscopy, biomedical optics, fluorescence, confocal optics, *in vivo* pathology

## Abstract

Current diagnostic capabilities and limitations of fluorescence endomicroscopy in the cervix are assessed by qualitative and quantitative image analysis. Four cervical tissue types are investigated: normal columnar epithelium, normal and precancerous squamous epithelium, and stromal tissue. This study focuses on the perceived variability within and the subtle differences between the four tissue groups in the context of endomicroscopic *in vivo* pathology. Conclusions are drawn on the general ability to distinguish and diagnose tissue types, on the need for imaging depth control to enhance differentiation, and on the possible risks for diagnostic misinterpretations.

## 1 Introduction

In high-resource settings, screening for cervical precancer and cancer is commonly performed by routine Pap smear cytology and may be accompanied by DNA testing for human papillomavirus (HPV) infections. Patients with high-risk HPV infections and/or abnormal Pap smear results are usually referred for colposcopy of the cervix which provides a low-magnified vision onto the cervical epithelium. Application of acetic acid on the cervix is used to identify areas of high nuclear density and application of Lugol’s iodine is used to identify both areas of abnormal glycogen content and the squamo-columnar junction (SCJ) with normal squamous epithelium being glycogenated.^1^ While Pap smear screening is associated with low sensitivity (53%) and high specificity (96%) for the detection of precancerous lesions,^2^ colposcopy alone (i.e., without biopsy evaluation) has a higher sensitivity (85%) and a rather low specificity (69%).^3^ In most cases, tissue biopsies are sampled during colposcopy to ascertain preliminary diagnosis and to decide upon the necessity for tissue resection using a loop electrosurgical excision procedure (LEEP). Thus, in spite of being the established methods for cervical cancer screening, colposcopy and Pap smear cytology are limited in their diagnostic capabilities. In contrast, colposcopically directed biopsies provide a definitive assessment of the collected tissue and represent the current gold standard in diagnosis of cervical precancers. However, the limited number of biopsies can fail to sample a precancerous area on the cervix and many biopsies may be taken unnecessarily from healthy tissue.^4^ Therefore, colposcopists find themselves in the conflict between diagnostic yield and the invasiveness of additional biopsies.

Fluorescence confocal endomicroscopy has the potential to provide clinicians with the ability to assess tissue grade by generating real-time cellular-level images of morphological features. The attainable contrast and resolution have already been demonstrated for cervical,^5^^–^^7^ oral,^8^^–^^10^ and ovarian^11^ tissues in numerous studies. With immediate access to this additional information, clinicians may arrive at definitive diagnosis even without histopathologic assessment.^12^ Such *in vivo* pathology may spare unnecessary tissue sampling and associated costs to the system as well as discomfort to the patient. Furthermore, it may provide the patient with immediate diagnosis and treatment options at the same time.^13^

Over the past 15 years not only fluorescence but also reflectance endomicroscopy^14^^–^^18^ have been extensively investigated as a means for *in vivo* pathology and tissue differentiation. Reflectance microscopy relies on cellular and especially nuclear light backscattering.^19^^,^^20^ Due to the forward directedness of light scattering in tissue, it suffers from low light yield and associated low imaging contrast. In comparison, fluorescence microscopy benefits from the isotropic emission of fluorescence light which originates from exogenous DNA-staining fluorophore molecules. A dichroic mirror allows for efficient separation of excitation and emission light and thus good contrast and comparably high image quality may be achieved.^21^^–^^24^ The high imaging contrast of fluorescence endomicroscopy is paired with a limited imaging depth of about 50  μm owing to the finite permeation of the fluorophore dye.^25^

Previous studies have successfully demonstrated how endomicroscopic imaging of the cervix can provide nuclear and coarse cellular morphology, which, when evaluated quantitatively^15^^,^^19^^–^^21^ or qualitatively,^5^^,^^20^ may be correlated with histology and precancerous states. In order for cervical *in vivo* microscopy to be used as an independent diagnostic tool for biopsy guidance and beyond, it is a prerequisite to thoroughly understand its structural contrast and its diagnostic limitations for all tissue types that are commonly observed in the cervix. Therefore, this work presents a fluorescence confocal endomicroscopy setup and uses both *in vivo* and *ex vivo* cervical imaging data to draw conclusions on its capabilities and challenges for *in vivo* diagnosis. By comparing *en face* endomicroscopic cervical images with coregistered stained histology sections, we study typical structural features of squamous epithelium, columnar epithelium, and stromal tissue. In contrast to previous studies, we also place special focus on the observed variability within each tissue type and the potential risk to misinterpret normal or inflammatory endocervical epithelium or stroma as precancerous high grade squamous intraepithelial lesions (HSIL) including cervical intraepithelial neoplasia 2 (CIN 2) and CIN 3.

The SCJ of the cervix is where squamous and columnar epithelium meet. Over the lifetime of a woman, the location of the SCJ shifts and the columnar cells are covered by and then replaced with squamous cells in a normal process called metaplasia. This area of transition where metaplasia occurs is called the transformation zone and is most susceptible to HPV infection and the development of precancer and cancer. Consequently, effective cervical cancer screening has to sample this intersection between columnar and squamous epithelium. When assessing fluorescence endomicroscopy as a potential *in vivo* diagnostic tool, it is, therefore, necessary to study the imaging characteristics of both columnar and squamous epithelium. The endomicroscopic identification of both columnar epithelium and stromal tissues is especially meaningful as the SCJ is not always circular and visible.

In Sec. 2, we present the experimental details of our imaging setup along with the tissue imaging protocol. Section 3 presents image analysis performed to determine the endomicroscopic differentiability of the four relevant diagnostic tissue types: normal squamous epithelium combined with low-grade squamous intraepithelial lesions (LSIL), HSIL, normal columnar epithelium, and stromal tissue. Adenocarcinoma *in situ* (AIS) could not be considered as an additional diagnostic group in our analysis because there were too few cases. Furthermore, AIS is sometimes hidden in occluded glandular structures or inaccessible for imaging inside the endocervical canal, which undermines definitive histopathologic coregistration.

Our image analysis focuses on structural image features which are commonly used to distinguish the different tissue types, and we quantitatively investigate the diagnostic capabilities and limitations of *in vivo* confocal fluorescence endomicroscopy in Sec. 4.

## 2 Imaging Setup and Procedure

Our imaging system is designed to perform *in vivo* fluorescence endomicroscopy in the cervix using the exogenous fluorophore acriflavine hydrochloride (Sigma-Aldrich, St. Louis, Missouri). This dye was diluted to a concentration of 0.05% using a 0.9% solution of sodium chloride (saline). Before imaging, the fluorophore was topically administered onto the cervical mucosa and about 10 s after application, residual stain was rinsed with saline.

**Fig. 1:**
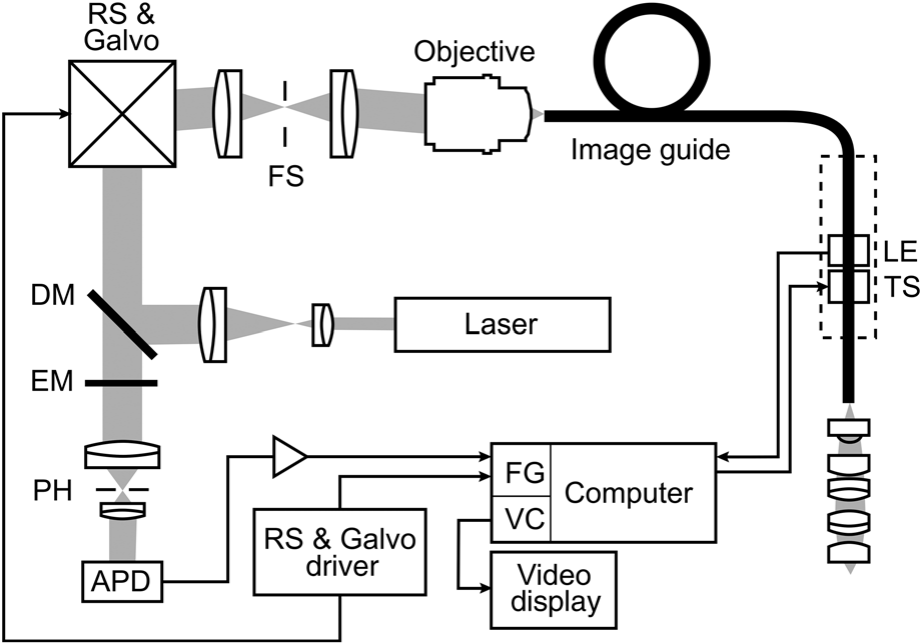
Schematic diagram of the imaging system. Laser light is reflected by a dichroic mirror (DM) and scanned by a resonance scanner (RS) and galvanometer (Galvo) over a field stop (FS) which is reimaged onto the proximal end of a fiber-optic image guide. An emission filter (EM) blocks reflected excitation light from detection by the avalanche photo diode (APD) placed behind a detection pin hole (PH). frame grabber (FG) and video card (VC) establish images and display. A piezo translation stage (TS) and linear encoder (LE) allow for changing and monitoring of the axial focus.

Figure 1 shows our experimental setup. Fluorophores are excited near their absorption maximum using a laser diode module (Cube 445-40C, 40 mW, Coherent, Santa Clara, California) at a wavelength of λ=445  nm.^26^ The projection optic produces a small laser spot size which is further reduced by the many projection fibers acting like projection pinholes. Confocality is ensured by a detection pinhole of 50  μm in diameter. Laser power as measured at the tip of the probe is 0.3 mW. Excitation light is coupled into the excitation–emission path by a dichroic mirror. A resonant scanner (CRS, GSI Group, Bedford, Massachusetts) and a galvanometer (VM500, GSI Group, Bedford, Massachusetts) scan the excitation point source over the fibers of an image guide (FIGH-30-650S, Myriad Fiber Imaging, Massachusetts). The image guide is composed of 30,000 individual fibers and light is coupled into the fibers using a multi-immersion microscope objective lens (Nikon, 20×/0.75, Tokyo, Japan). Index matching oil of refractive index 1.492 (Cargille Laboratories, Cedar Grove, New Jersey) is used between the image guide and objective lens to closely match the refractive index of the fiber cores. The image guide carries the excitation signal to the imaging site and a chromatically corrected lens assembly at the distal end of the image guide projects the excitation and detection light to and from the tissue. The distal objective lens has a magnification of 3× thereby producing an imaging field of view of 240  μm in diameter with lateral and axial resolutions of 1.3 and 10  μm, respectively. The lateral resolution is limited by the fiber spacing of the image guide (3.3  μm between neighboring fiber cores), whereas the NA of the imaging guide (0.35) limits the numerical aperture at the tissue to 1.05.

Acriflavine is a DNA stain that temporarily intercalates nucleic acids. Consequently, the administered fluorophore accumulates predominantly in the nucleus of cells and fluorescent light that is in axial focus passes both the dichroic beam splitter and the detection pinhole. An avalanche photodiode (C5460, Hamamatsu, Bridgewater, New Jersey) captures the scanned fluorescence emission and a frame grabber (Meteor II, Matrox, Dorval, Quebec) both amplifies and digitizes the detected light and generates images by use of synchronization signals from the resonant scanner. A video frame rate of 7  frames/s is thereby obtained and displayed in near real time.

**Fig. 2:**
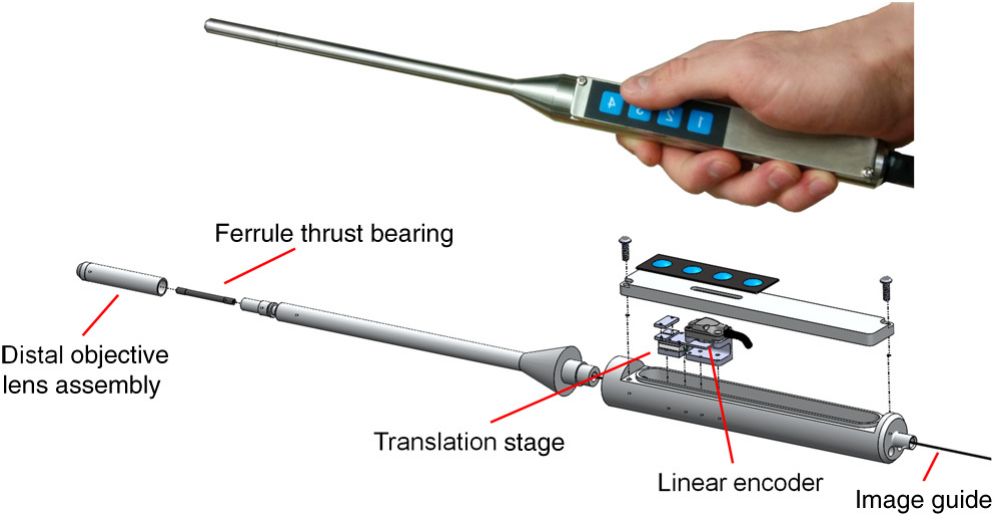
The handheld wand and its internal assembly. By use of a translation stage, the image guide is allowed to glide back and forth with respect to the distal lenses thereby enabling depth focusing. An linear encoder provides feedback on the position of the fiber bundle. The diameter and length of the distal portion of the handheld wand measure 9 mm and 19 cm, respectively.

The employed image guide is 1.4 m long and thus allows for imaging tissue both *ex vivo* and *in vivo*. The possibility to image at various tissue depths is achieved by actuating the image guide relative to the distal lens assembly using a piezo linear stage (Micronix PP-12, Irvine, California). A linear encoder (MTE, Micro-E Systems, Bedford, Massachusetts) tracks the imaging depth at a resolution of 5  μm and allows for quantified optical sectioning control. Both the linear stage and the linear encoder are built into the handheld wand, which is presented in Fig. 2.

Every imaging procedure starts with zeroing of the depth scale by scanning to the uppermost tissue layer that comes into focus. In doing so, absolute depth scaling is obtained for subsequent image captures. Our imaging device is controlled using custom software which allows for: changing the focal depth, recording videos and images, basic image corrections, and contrast enhancement.

The imaging data evaluated for this study comprise *ex vivo* imaging on cervical tissue specimens of 58 women (16 biopsies and 42 LEEPs) and *in vivo* cervical imaging on 4 women. Table 1 shows a summary of patient characteristics in terms of number of patients in this study, tissue specimen type, patient ages, patient diagnoses, and the volume of imaging data. As part of the video recording, numerous depth scans (video stacks) were performed without moving the probe laterally, thus capturing contrast from different tissue layers. It should be noted that all four patients imaged *in vivo* turned out to be negative for dysplasia.

**Table 1:** Characteristics of the studied tissue specimens.

Patients	62
*Ex vivo* biopsies	16
*Ex vivo* LEEPs	42
*In vivo*	4
Age	21 to 50 (mean 32)
Diagnoses	
Negative for dysplasia	6
HPV/CIN 1	15
CIN 2	26
CIN 3/CIS	27
Invasive squamous cancer	0
AIS	6
Invasive adenocarcinoma	0
Image data reviewed	
Videos	≈500 (14 h)
Video stacks (depth scans)	352

Informed consent was obtained from all patients and the imaging protocol was approved by the Ethics Board of the British Columbia Cancer Agency under the ethics numbers H11-00011 and H09-03303. For all *ex vivo* cases, image recording was performed within 1.5 h of biopsy resection or LEEP. In succession to microscopy on multiple imaging locations, tissue samples were immersed in formalin and forwarded to histopathology. High-resolution haematoxylin and eosin (H&E) stained histology images of vertical tissue sections are then compared to the *en face* endomicroscopy images. Topical green inking of imaged tissue locations allowed for definite coregistration of endomicroscopic images with histology sections that carried the green ink. The subsequent image analysis and interpretation was performed in consideration of the diagnosis provided by a board certified pathologist.

For tissue imaging, the handheld wand was covered by a plastic sheath. Furthermore, a three stage instrument cleaning and sterilization protocol was used before *in vivo* imaging. This protocol involved successive wiping and soaking of the probe with enzymatic solution (Endozime AW, Ruhof, Mineola, New York) followed by two different disinfecting agents (Cidex OPA, Cidex, Irvine, California; Cavi Wipes, Metrex, Orange, California).

## 3 Imaging Results and Analysis

In the following, we present four selected fluorescence endomicroscopy images for each of the four tissue categories along with correlated H&E-stained histology sections of the same tissue sample from a nearby location. Successful histological coregistration was enabled by topical green inking of the imaging location after imaging and before formalin fixation on every LEEP tissue sample. For each of the studied tissue categories, we discuss the variation in endomicroscopic appearance which we frequently observe throughout all investigated tissue. This allows us to both name the subtle imaging features that are relevant for tissue differentiation and to determine the risk for diagnostic misinterpretation.

### 3.1 Normal Squamous Epithelium (Including LSIL)

LSIL, also denoted as cervical intraepithelial neoplasia 1 (CIN 1), are considered to be lesions of the cervix that do not require clinical intervention. Epithelial transformations associated with LSIL are confined to the lower part of the squamous epithelium and are not detectable by fluorescence endomicroscopy.^27^ For both reasons, we do not discriminate between normal squamous epithelium that is free of dysplasia and LSIL in this study.

**Fig. 3:**
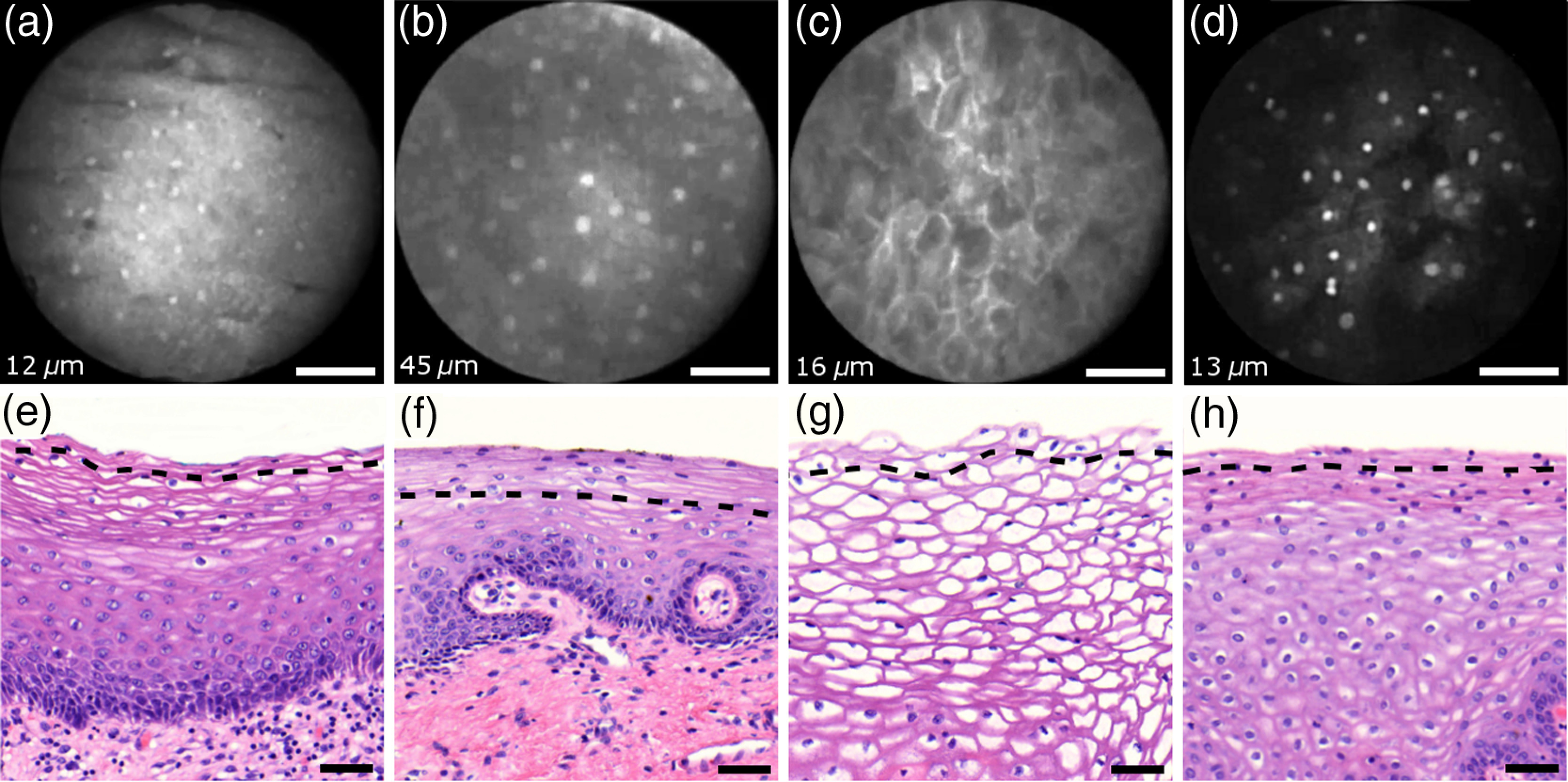
Normal squamous epithelium as imaged by endomicroscopy (a)–(d) along with coregistered histological sections (e)–(h). Scale bars measure 50  μm. Dashed lines indicate the corresponding imaging depth as quantified in (a)–(d). Image (c) and section (g) depict glycogen rich epithelium. Image (d) was captured *in vivo*.

The appearance of normal squamous epithelium in endomicroscopy and histology is illustrated in Figs. 3(a)–3(d) and 3(e)–3(h), respectively. Both low nuclear density (i.e., small nucleus-to-nucleus spacing) and small nuclear size have been established as important parameters for differentiating normal squamous epithelium from HSIL in the cervix.^5^^,^^21^ Endomicroscopic images in Figs. 3(a)–3(d) all feature low nuclear density. However, the apparent nuclear size in these four images differs considerably and so does the imaging depth as stated in Figs. 3(a)–3(d). This imaging depth is also marked in the H&E sections of Figs. 3(e)–3(h) by a dashed line. It should be noted that histological sections presented in Fig. 3 and subsequent figures display uncorrected scaling without accounting for possible tissue shrinkage by formalin fixation.^27^

Figure 3(a) shows small, pyknotic, and widely separated nuclei, which is one of the most common endomicroscopic appearances of well-differentiated normal squamous epithelium. Figure 3(b) has been captured at a comparatively greater depth of 45±5  μm, which—depending on the overall epithelial thickness—increases the chance of seeing less differentiated cells with larger nuclei. The different brightness of nuclei in 3(b) indicates that cells of different layers contribute to the image contrast which may lead to a perceived higher nuclear density in stratified epithelium.

Depending on the phase of the menstrual cycle, squamous epithelium contains a variable amount of glycogen. Figures 3(c) and 3(g) show epithelial cells with an enlarged cytoplasm as caused by a buildup of glycogen. The cytoplasmic enlargement leads to an increased cell thickness. In consequence, lateral cell boundaries start to extend throughout the axial focus thus enhancing their visibility and promoting single-cell differentiability as can be observed in 3(c). The enlightened cell boundaries can either be explained by residual fluorophores in the extracellular matrix or by some staining effect of acriflavine on cellular membranes or extracellular molecules.

Figure 3(d) shows normal squamous epithelium which was captured *in vivo*. *In vivo* images tend to demonstrate similar image contrast as resected *ex vivo* specimens. We observe that fluorophore dye penetration is enhanced for tissue biopsies, which can be explained by their small size and incomplete epithelial lining. Conversely, diminished dye penetration may explain the strongly reduced background fluorescence in Fig. 3(d). While background fluorescence generally still tends to be present *in vivo*, we did not observe any *ex vivo* samples with levels as low as depicted. Beyond differences in dye permeation, *in vivo* and *ex vivo* endomicroscopy give rise to similar image contrast with the increased challenge of motion stability *in vivo*.

### 3.2 High-Grade Squamous Intraepithelial Lesions

**Fig. 4:**
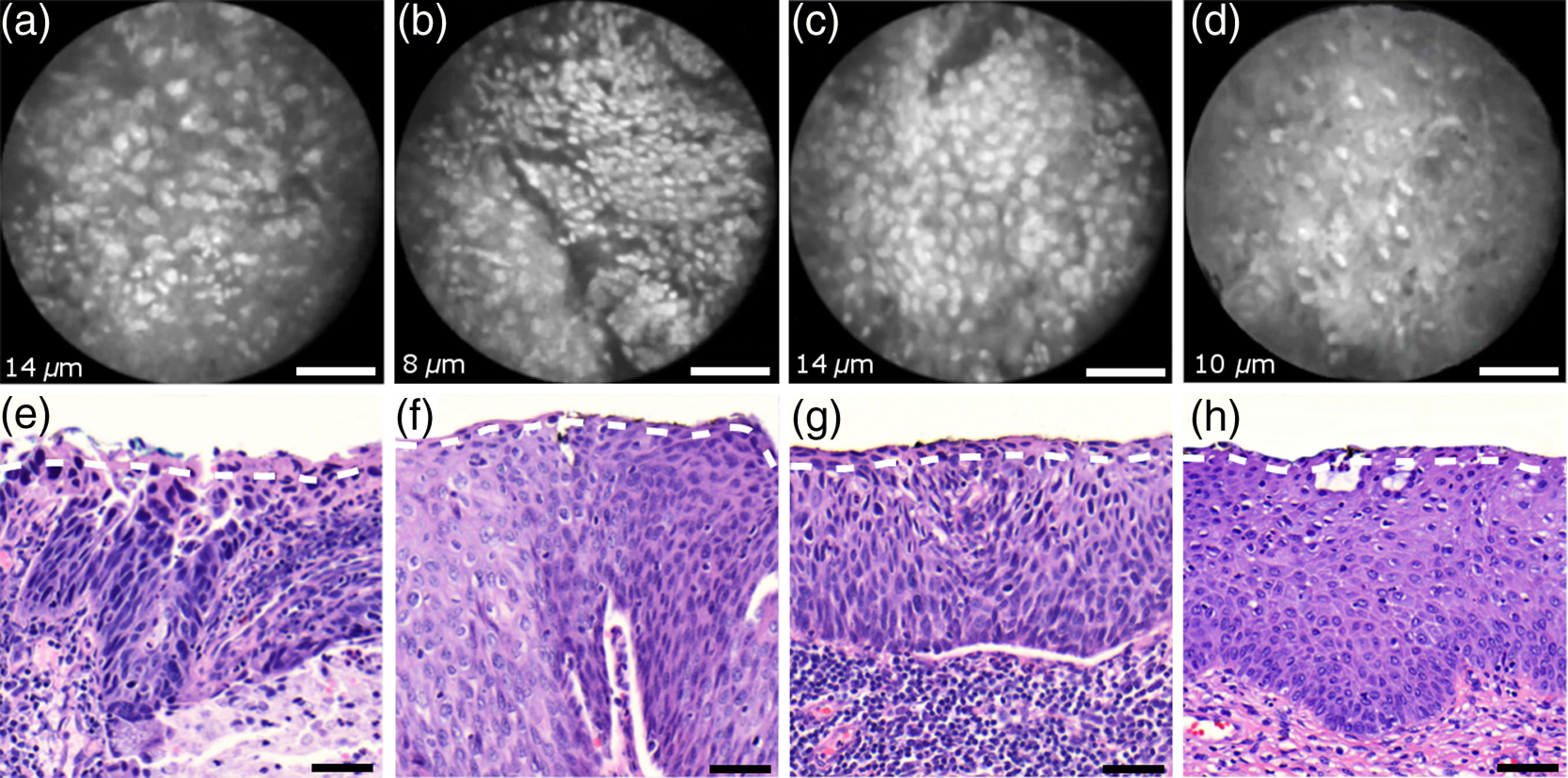
Endomicroscopy of HSIL as captured at superficial depths (a)–(d) and stained histology sections (e)–(h) corresponding to the same imaging locations. Scale bars measure 50  μm. (d) and (h) is classified as CIN 2, while all other images contain CIN 3 tissue.

Figure 4 shows imaging on the precancerous squamous epithelium, or HSIL, which represents the second tissue category in our study. All four endomicroscopic images in Figs. 4(a)–4(d) were captured *ex vivo* and depict high nuclear density, large nuclei, and a correspondingly high nuclear to cytoplasmic ratio. The latter is not entirely true for Fig. 4(d) which corresponds to a less advanced HSIL tissue state (CIN 2) captured at a depth of 10  μm.

In accordance with previous studies, the presence of high nuclear density in superficial squamous epithelium is a hallmark of preinvasive disease.^5^^,^^21^ By comparing the endomicroscopic imagings of Figs. 3 and 4, it seems obvious that marked and systematic differences between normal and high grade dysplastic squamous epithelium exist. Figures 3(b) and 4(d) show similarities in nuclear size and spacing and the subtle differences between the two images capture the challenge in correctly detecting and interpreting CIN 2 lesions. Their proper interpretation and comparison should, however, take the differences in imaging depth into consideration. Figure 4(d) shows the uppermost epithelial cell layer, whereas Fig. 3(b) shows imaging at a depth of 45±5  μm where evenly enlarged nuclei may belong to less differentiated yet benign epithelial cells depending on the overall thickness of the epithelium. It is worth noting that unambiguous grading of CIN 2 lesions is often found to be challenging even in histopathology.^28^ With respect to proper CIN 2 differentiation in endomicroscopy, one may appreciate the utility of varying the absolute imaging-depth, as quantified in the endomicroscopic images of Figs. 3 and 4.

**Fig. 5:**
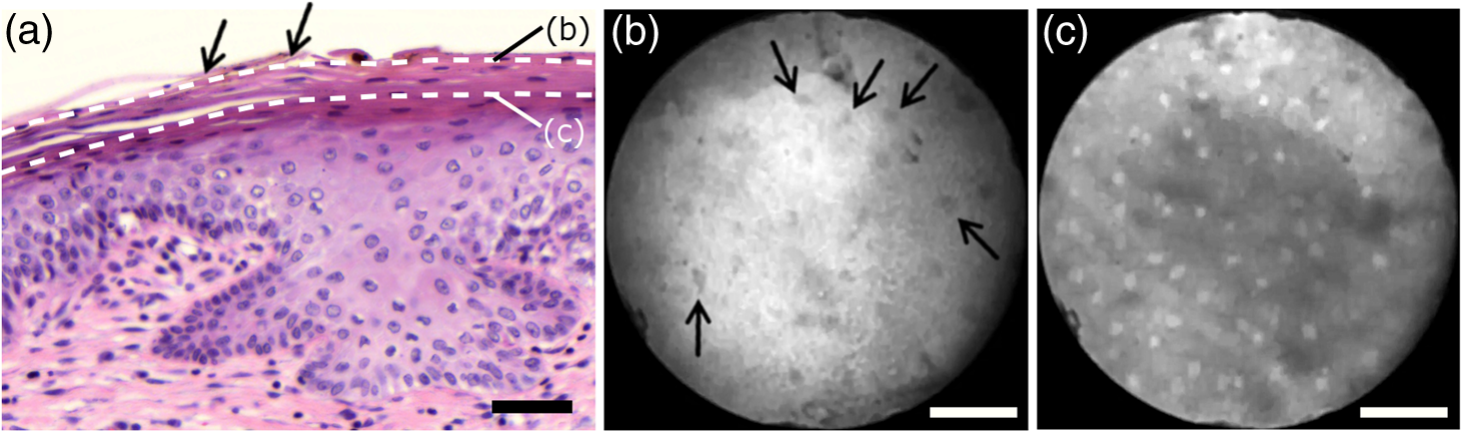
Demonstration of the effect of autofluorescence on fluorescence endomicroscopy of cervical squamous epithelium. Section (a) shows a histological tissue section with traces of keratinization. Images (b) and (c) depict corresponding endomicroscopic images that were captured at different epithelial depths. Superficial autofluorescence undermines the visibility of top layer nuclei in (b).

In addition to our general investigations on nuclear structure and contrast in squamous epithelium, we also observed endogenous epithelial fluorescence from within squamous epithelium. Throughout all our investigated *in vivo* and *ex vivo* imaging data, we occasionally find superficial squamous epithelium to give rise to an unusually large fluorescence signal that mostly lacks nuclear contrast. We attribute this effect to tissue autofluorescence which might have its origin in keratin fibers and cellular keratinization. Figure 5 shows an example of this observation, with the histological H&E section in 5(a) indicating traces of keratinization in the top orange staining epithelial tissue layers (see arrows). Figures 5(b) and 5(c) show two corresponding endomicroscopic images of a nearby tissue location. The two images were captured at two different imaging depths which are indicated by dashed lines in the histological section in Fig. 5(a). Accordingly, Fig. 5(b) was captured from a superficial cell layer and resolves no fluorescening nuclei. This image contains numerous dark spots which most likely result from absorption of fluorescence emission by superficial red blood cells [see arrows in 5(b)]. Figure 5(c) was captured at a deeper cell layer and is less affected by the strong superficial autofluorescence signal. Stained nuclei exhibit stronger contrast compared to background autofluorescence at this deeper layer.

### 3.3 Normal Columnar Epithelium

**Fig. 6:**
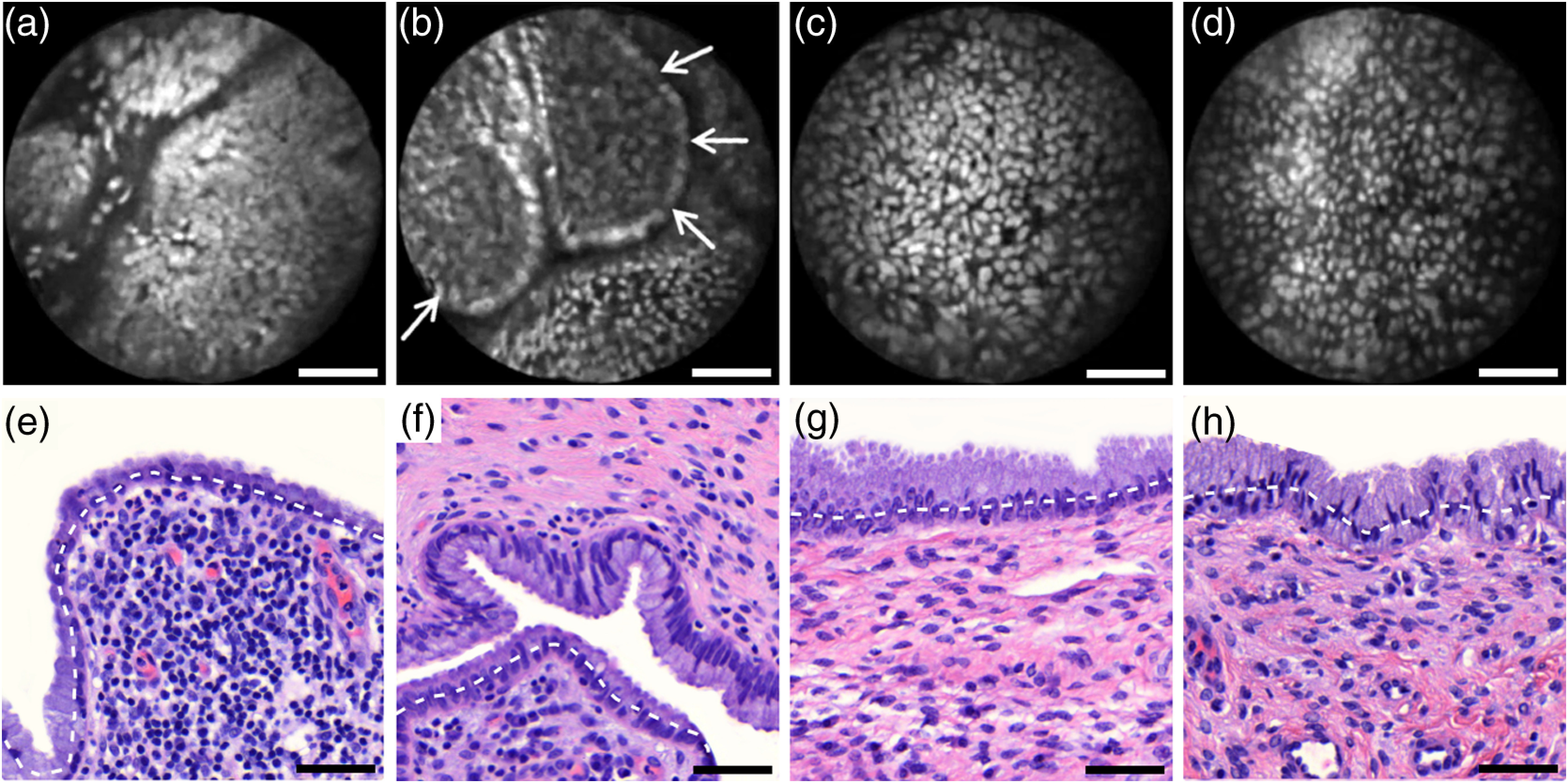
Endomicroscopic images (a)–(d) and coregistered H&E stained histological sections (e)–(h) of normal columnar epithelium. Scale bars measure 50  μm. The focus of all four endomicroscopy images is set to image the uppermost columnar cell layer. Approximate imaging depths are indicated by dashed curves in every histological section.

Figure 6 shows endomicroscopic images [(a)–(d)] and corresponding histological sections [(e) and (f)] of normal columnar epithelium. For Figs. 6(a)–6(d), no absolute imaging depth values were recorded; however, all four images represent the outermost tissue layer that came into focus during *ex vivo* tissue endomicroscopy and thus exclusively depict columnar epithelium. We expect the imaging depth of all four images to be in the range of 9 to 18  μm, as inferred from imaging features and both endomicroscopic and histological sectioning. Again, the estimated imaging depth of Figs. 6(a)–6(d) is marked in the four corresponding histology sections in Figs. 6(e)–6(h) by dashed lines.

Stable and in-focus imaging of columnar epithelium can be challenging as its undulating and ductile structures can easily give rise to small drifts upon slight variation of contact pressure from the imaging wand. The wavelike surface structure may at the same time serve as a useful indicator for columnar epithelium, as observable in Figs. 6(a) and 6(b).

Figures 6(a)–6(d) show different appearances of columnar epithelium. By analysis of the corresponding histological sections 6(e)–6(h), the strong variability in shape, size, and polarity of epithelial nuclei and cells becomes apparent. This structural variation causes inconsistent appearance in *en face* endomicroscopy. In Fig. 6(a), nuclei appear densely packed due to the round nuclear shape that is observable in the columnar epithelium in Fig. 6(e). Note that the densely packed inflammatory cells (lymphocytes) underneath the columnar epithelium in Fig. 6(e) are not imaged due to the superficial focus of Fig. 6(a).

Figure 6(b) shows bright columnar cells (see arrows) which seem to enclose rounded tissue sections. These bright cells are focally aligned along the folds of the undulating tissue structure corresponding to the indicated imaging depth (dashed line) in Fig. 6(f). The centers of these bounded areas also represent columnar nuclei, which are located more superficially and are thus only partly in focus, less bright, and seemingly less dense. Owing to the axial resolution of about 10  μm, fluorescence from multiple nuclear layers may sometimes be captured at the same time.

In the absence of apparent ductile tissue structures, Figs. 6(c) and 6(d) also show columnar tissue with the presence of high nuclear density. Differences in nuclear polarity within the columnar cells [compare 6(g) and 6(h)] and variation of the focal plane relative to the nuclei in the columnar epithelium can lead to differences in perceived nuclear spacing in the *en face* image.

In conclusion, we observe strong variability in the appearance of columnar epithelium in fluorescence endomicroscopy along with differences in perceived nuclear size owing to the different shapes and alignment of nuclei. Figures 6(a)–6(d) show nuclear crowding similar to that observed in Fig. 4. While the presence of ducts and tissue folds may hint at columnar tissue, it is very challenging to come up with clear-cut features for differentiation of normal columnar epithelium and HSIL. Imaging at multiple depths and analysis of the variation in nuclear size and mutual alignment may assist in the differentiation of columnar tissue. However, we observe a decreased maximum imaging depth in areas of columnar epithelium, explicable either by limited permeation of dye beyond the basement membrane or by the strong signal from within the columnar epithelium.

### 3.4 Stroma

Stromal tissue is usually covered by columnar or squamous epithelium, but is sometimes denuded in metaplastic areas within the transformation zone or at sites of previous biopsy. It is, therefore, useful to understand the stromal appearance in endomicroscopy to allow for its visual discrimination from epithelial tissue.

**Fig. 7:**
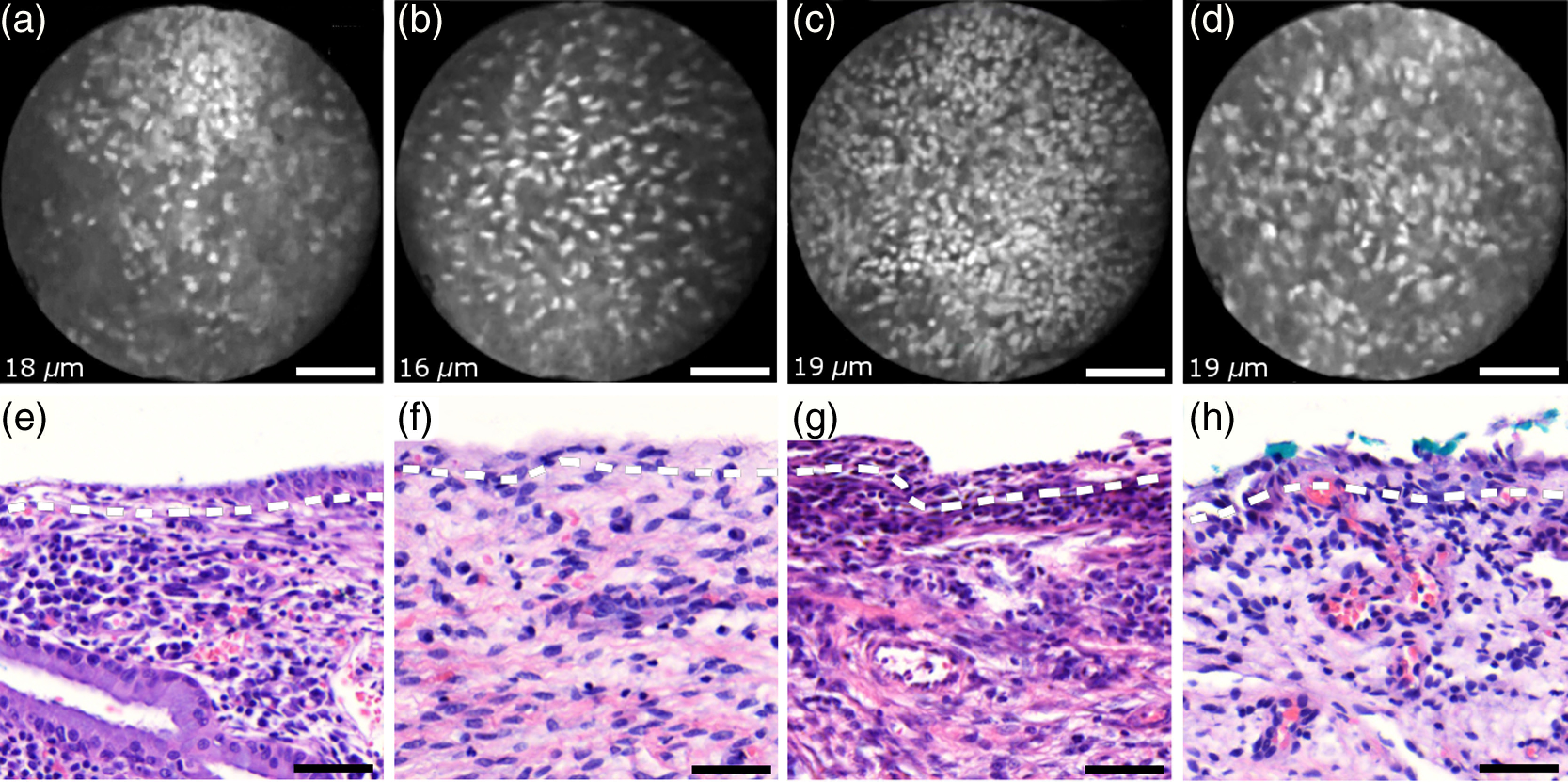
Endomicroscopy and histology of stromal tissue. Images (a)–(d) represent en face endomicroscopic appearance and sections (e)–(f) display the corresponding tissue locations after histological cross sectioning and H&E staining. Scale bars are 50  μm and dashed lines indicate the absolute imaging depth provided with images (a)–(d).

Figures 7(a)–7(d) show microscopy from four stromal tissue imaging sites of different tissue samples. Again, imaging locations could be successfully coregistered with corresponding histological sections in Figs. 7(e)–7(h) by the use of green ink. Some of such green ink markings are visible on the stromal surface of Fig. 7(h). The imaging depth is stated in each endomicroscopic image and was for all four images recorded to be (16−19)±5  μm, which corresponds roughly to the second or third stromal cell layer.

Both the small and round lymphocytes, which are particularly dense in sections in Figs. 7(e) and 7(g), and the elongated nuclei of stromal cells [see sections in Figs. 7(f) and 7(h)], are stained by acriflavine and thus visualized in endomicroscopy. A characteristic feature of stromal tissue is the seemingly randomized alignment of stromal nuclei. This is in contrast to superficial cell layers in dysplastic stratified squamous epithelium. Figures 7(b) and 7(d) show various projections of stromal nuclei which give the visual impression of disorder owing to the different orientations of small but elongated nuclei.

The inflammatory tissue in 7(a) and 7(c) is apparent by the high nuclear density in combination with very small but round nuclei. It is worth noting that cervical precancer is very often accompanied by densely packed inflammatory cells in proximal stroma. In contrast, the inflammatory tissue depicted in Fig. 7(e) corresponds to an LEEP sample which was found to be negative for dysplasia.

**Fig. 8:**
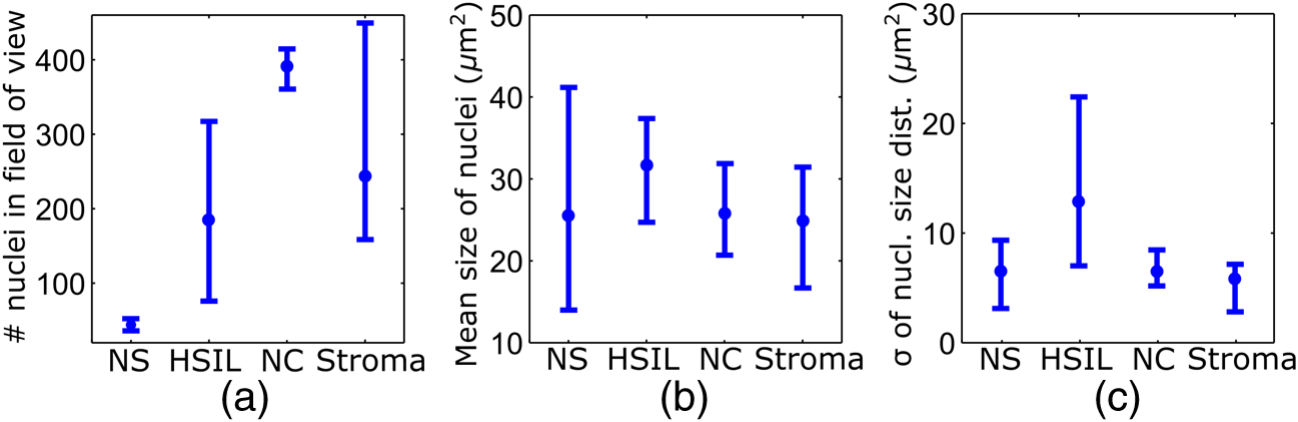
(a) Evaluation of nuclear density, (b) mean nuclear size, (c) and the standard deviation of the nuclear size distribution for the presented images of the different tissue types (NS, normal squamous, NC, normal columnar). Data bars quantify the minimum, mean, and maximum values.

In support of our qualitative observations, Fig. 8 shows a basic quantitative evaluation of all presented fluorescence images in Figs. 3, 4, 6, and 7. Figure 8(a) shows the total number of nuclei in the imaging field of view for all four investigated tissue types. Every bar quantifies the minimum, mean, and maximum value as determined from the four corresponding images of each category. Figures 8(b) and 8(c) show the mean size and the standard deviation σ of the nuclear size distribution, respectively. Again, the four corresponding images of each category were studied independently and the bars in Figs. 8(b) and 8(c) quantify the minimum, mean, and maximum value of the image ensemble.

Figure 8 shows that neither nuclear density [Fig. 8(a)] nor the mean size of nuclei [Fig. 8(b)] can easily distinguish HSIL from the other three tissue types. However, we find that the standard deviation σ of the nuclear size distribution tends to be noticeably higher for high-grade squamous lesions [Fig. 8(c)]. This can be explained both by a lack of cellular differentiation in HSIL and by a relative overall increase in nuclear size.

## 4 Stack Analysis

An important novelty of our imaging setup is its ability to vary the focal depth. In consequence, we endeavor to understand the value of this added feature over traditional imaging, typically performed at a fixed superficial depth where contrast and signal intensity are at their optimum. To address this question, a group of five trained individuals (four research scientists and one clinician-scientist) was recruited to assess a set of 352 video stacks containing vertical scans and to compare each stack to a single image from one of the uppermost cellular layers. The five observers were trained on the images from Figs. 3, 4, 6, and 7 to discriminate the four tissue types. Stacks and corresponding single frames were compared with respect to the perceived impression of tissue type and diagnosis. Video stacks are generally limited to imaging of the uppermost two to three cell layers owing to the penetration of fluorophores.

Observers who completed this comprehensive stack evaluation found that variable depth focusing was helpful and changed their impression of the tissue on average in 20% (3%, 17%, 20%, 29%, and 29%) of the 352 cases. The stack analysis comprised imaging data of all previously discussed tissue types. The large majority of cases where observers found the stacks to be helpful involved distinguishing among HSIL, columnar, and stromal tissues.

In order to assess how well confocal endomicroscopy works for identifying and differentiating cervical tissue types, all five observers were asked to independently group every image-stack pair into one of four diagnostic groups: normal squamous (including LSIL), HSIL, normal columnar, and stroma. If observers found themselves unable to make a decision based on insufficient image quality, then they were allowed to exclude the case from their analysis.

Table 2 shows our evaluation results. Accordingly, all five observers unanimously agreed in their diagnostic categorization in 39.6% of all cases (note that we evaluated only those stack-image pairs which were not excluded by any observer, i.e., 283 cases). Agreement was especially high for normal squamous epithelium. Out of all cases where at least one observer diagnosed “normal squamous” (177 cases), unanimity was reached by a relative probability of 51%. By contrast, agreement among all observers on the diagnosis “HSIL” with respect to all cases where at least one observer diagnosed “HSIL” (150 cases) turned out to be only 12%. Unanimous diagnosis for columnar or stromal tissue was rarely achieved for the investigated cases.

**Table 2:** Agreement of five trained observers in their diagnostic classification of stack-image pairs. For each diagnostic category, we include all cases where at least one observer made the corresponding diagnosis. In 283 cases, all five observers provided a diagnosis.

	Observer unanimity (%)	Number of cases associated with group
Normal squamous (+LSIL)	51.4	177
HSIL	12.0	150
Normal columnar	3.3	91
Stroma	0.0	81
Total	39.6	283

It is worth mentioning that in 38% of cases out of the 150 cases associated with the “HSIL” group all five observers had selected either “HSIL” or “normal columnar” as their diagnosis. This underlines the similar appearance of normal columnar and precancerous squamous epithelium in fluorescence endomicroscopy and thus the challenge of correct diagnostic differentiation.

## 5 Conclusions

Cervical *in vivo* confocal microscopy has the potential to provide clinicians with real-time cellular-level images for evaluation of morphological and architectural features. As such, it may be readily employed as an adjunct to colposcopic examination to facilitate proper biopsy-site selection. In this respect, our qualitative analysis provides a reference for interpretation of common endomicroscopic imaging features.

If the usage of *in vivo* microscopy could be extended from mere biopsy guidance toward an independent tool for instant and accurate diagnosis, its clinical benefit could be vastly enhanced. In this regard, our study serves as a general assessment of the imaging capabilities of cervical fluorescence endomicroscopy, which at the same time reveals challenges for independent *in vivo* pathology. The conclusions drawn from the presented images of squamous, columnar, and stromal cervical tissues represent the comprehensive observations made on a large set of imaging material from over 60 patients.

As was shown in the previous studies, HSIL and CIN 3 in particular can be readily distinguished from normal squamous epithelium using fluorescence microscopy.^5^^,^^21^ Our analysis confirms this promising result while creating awareness for the occurring structural similarities of HSIL and both stromal and columnar tissues in *en face* endomicroscopy. When taking all four tissue categories into consideration, reliable differentiation by fluorescence endomicroscopy alone may be very challenging. This is underlined by the low interobserver agreement in our impression-based data analysis. Nevertheless, given the possibility to demarcate squamous and columnar epithelium using Lugol’s iodine, it may well be possible to reach unambiguous diagnosis of HSIL using fluorescence endomicroscopy in a majority of cases.

Furthermore, a more quantitative analysis of captured images may make the delineation easier, as the simultaneous presence of high nuclear density along with large nuclear variability in size and shape may be strongly associated with HSIL.^21^ In HSIL, nuclear crowding often leads to the mutual alignment of neighboring nuclei and thus aids in its visual discrimination. Large nuclei are rarely found in stromal tissue and columnar epithelium lacks local variability in nuclear size. Additionally, *in vivo* diagnosis of HSIL may often be facilitated by depth scanning, thus ruling out the presence of columnar epithelium that may have similar appearances in endomicroscopy.

Variation and absolute control of imaging depth is an asset for endomicroscopic systems given the need to differentiate normal squamous epithelium and HSIL, and to discriminate among columnar, squamous, and stromal tissue. An inherent limitation to the imaging system described is the penetration depth of extrinsic fluorophores, which restricts imaging to the uppermost cellular layers of the cervix and thus limits the possible gain in depth information. Nevertheless, this axial scanning capability has shown to provide additional information in 20% of cases and may also assist in the subtle differentiation between CIN 2 and normal squamous epithelium.

With the aspiration of highly reliable *in vivo* pathology, frequent challenges—may they be endogenous fluorescence or difficulties in tissue type delineation—will have to be overcome. In this regard, combinations with other techniques which feature an increased penetration depth or provide an additional form of contrast may add to the merits of cervical endomicroscopy.^29^^,^^30^ This could allow for even better and more intuitive discrimination of the presented four diagnostic categories including moderate level precancers (CIN 2) and potentially also AIS.
